# Automatic Lung Segmentation on Chest X-rays Using Self-Attention Deep Neural Network

**DOI:** 10.3390/s21020369

**Published:** 2021-01-07

**Authors:** Minki Kim, Byoung-Dai Lee

**Affiliations:** School of Computer Science and Engineering, Kyonggi University, Gyeonggi-do 16227, Korea; mkmk0612@kgu.ac.kr

**Keywords:** deep learning, medical image, attention module, image segmentation, lung segmentation

## Abstract

Accurate identification of the boundaries of organs or abnormal objects (e.g., tumors) in medical images is important in surgical planning and in the diagnosis and prognosis of diseases. In this study, we propose a deep learning-based method to segment lung areas in chest X-rays. The novel aspect of the proposed method is the self-attention module, where the outputs of the channel and spatial attention modules are combined to generate attention maps, with each highlighting those regions of feature maps that correspond to “what” and “where” to attend in the learning process, respectively. Thereafter, the attention maps are multiplied element-wise with the input feature map, and the intermediate results are added to the input feature map again for residual learning. Using X-ray images collected from public datasets for training and evaluation, we applied the proposed attention modules to U-Net for segmentation of lung areas and conducted experiments while changing the locations of the attention modules in the baseline network. The experimental results showed that our method achieved comparable or better performance than the existing medical image segmentation networks in terms of Dice score when the proposed attention modules were placed in lower layers of both the contracting and expanding paths of U-Net.

## 1. Introduction

Various studies on medical imaging using deep learning that combine medical imaging with image classification, detection, and segmentation have been conducted recently [1]. For example, ChexNet [2], which was developed by a Stanford University research team, demonstrated faster and more accurate identification of 14 chest X-ray-detectable diseases compared to specialists. In January 2020, DeepMind proposed an artificial intelligence model for breast cancer diagnosis. The model outperformed existing breast cancer diagnosis models by decreasing the false positive and false negative ratios by 3.5% and 8.1%, respectively, thus showing a lower breast cancer misdiagnosis rate than that of doctors [3]. Image segmentation is invaluable because it can distinguish specific interest areas by drawing boundary lines in an input image and identifying diseases by segmenting organs and tumors in medical images. For instance, Li et al. recently proposed a method that automatically identifies cardiomegaly by the cardiothoracic ratio, which is determined automatically using the image segmentation results of the lung and heart [4]. In such automatic disease identification systems, the performance of disease diagnosis is dependent on the image segmentation performance.

Deep learning is a type of feature learning that extracts and learns features from input images. Several researchers have constructed deep networks with considerable depths to extract meaningful features. In the ImageNet Large Scale Visual Recognition Challenge 2015, ResNet [5] triumphed with a top-five test error of 3.567% in the image classification division by using a network structure with a 152-network depth, which is eight times deeper than that of VGGNet [6]. By applying the residual module to a deep network, ResNet achieved a high top-five error performance that was 3.7% higher than that of VGGNet. However, a deeper network has more learning parameters, resulting in a lower learning speed and higher risk of overfitting. To address this problem, an attention module is being researched [7]. An attention module serves to highlight the values of important features, which are elusive during judgment, by emphasizing important features and removing features unnecessary for learning.

In this study, we propose a deep learning-based method for segmentation of lung areas from chest X-ray images. The novelty of the proposed approach is the self-attention module and its variant (e.g., X-attention module and Y-attention module), which makes use of the channel and spatial attentions that are extracted from the input feature maps. The proposed attention modules were applied to the standard U-Net [8] for validation. U-Net is a deep neural network structure that is frequently used in segmentation of medical images of various modalities such as X-rays, Magnetic Resonance Imaging (MRI), and Computed Tomography (CT). We conducted experiments to investigate the performance of the proposed deep learning-based lung area segmentation method.

In the next section, related works on lung image segmentation are introduced. Section 3 describes the proposed attention modules in detail. In Section 4, the experimental results obtained through a performance comparison of the proposed method with an existing medical image segmentation network are analyzed. Finally, the paper is concluded in Section 5.

## 2. Related Work

Several studies have been conducted on lung segmentation using conventional image processing techniques such as edge detection, threshold, and clustering [9]. However, the image processing methods have relatively simple algorithms and exhibit poor segmentation performance when the input image contains noise. With the development of deep learning, lung segmentation using convolutional neural networks is being actively researched. Currently, researchers are attempting to enhance lung segmentation performance not only by developing complex image segmentation network structures but also by using various techniques such as attention modules. In particular, the attention mechanism has demonstrated significant performance improvements in many deep learning-based tasks. Therefore, our review focuses on attention-based approaches for segmentation.

Attention U-Net [10] performed better than U-Net through the addition of an attention module with a simple structure between the contracting and expanding paths of the existing U-Net structure. Gusztav et al. [11] extended Structure Correcting Adversarial Network (SCAN) [12], which is the first attempt to use adversarial learning for lung segmentation on chest X-rays, by adopting Attention U-Net and Focal Tversky loss [13] for the generator network and its corresponding loss function, respectively. Furthermore, XLSor [14] used CC-Net [15] (which is an image segmentation network based on Criss-Cross Attention Module) as the backbone network and generated additional learning data using the image-to-image translation technique. XLSor is a state-of-the-art deep learning model for lung segmentation on chest X-ray images; thus, it has been used as an object of comparison for many lung image segmentation networks.

Since its introduction in SENet [16], channel attention has attracted significant research interest and proved its potential in improving the performance of deep neural networks. As examples of recent proposals, the Effective Channel Attention [17] module can learn effective channel attention by avoiding reduction in channel dimensionality while capturing cross-channel interaction in an extremely lightweight manner. The channel attention module proposed by DANet [18] exploits spatial information at all corresponding positions to model the channel correlations. The feature pyramid attention (FPA) module of Pyramid Attention Network (PAN) [19] fuses different scale context information through a pyramid structure to produce pixel-level attention for high-level features. It is similar to our attention modules in that both channel and spatial attentions are utilized to extract important features better. However, there are several differences in the network instantiation. For instance, we added the expanding path in the intermediate convolutional layers of the pyramid structure and adopted a residual learning scheme. In addition, our attention modules can be located in any of the layers in the segmentation networks. However, the FPA module is treated as the center block between the encoder–decoder structure. In the Recurrent Saliency Transformation Network [20], the segmentation probability map from the previous iterations is repeatedly converted into spatial weights that are, in turn, applied in the current iteration. This process enables multi-stage visual cues to be incorporated toward more accurate segmentation as well as joint optimization between a coarse-scaled network and a fine-scaled network. In particular, the saliency transformation function for adding spatial weights to the input image is based on a recurrent neural network.

A common characteristic across existing studies on lung segmentation is the absence of learning data wherein the contour of the lung is hidden or the lung shape is deformed. Consequently, existing methods achieve low segmentation performances for chest X-ray images containing hidden lung contours or deformed lung shapes.

## 3. Proposed Self-Attention Modules

### 3.1. X-Attention Module

**Figure 1:**
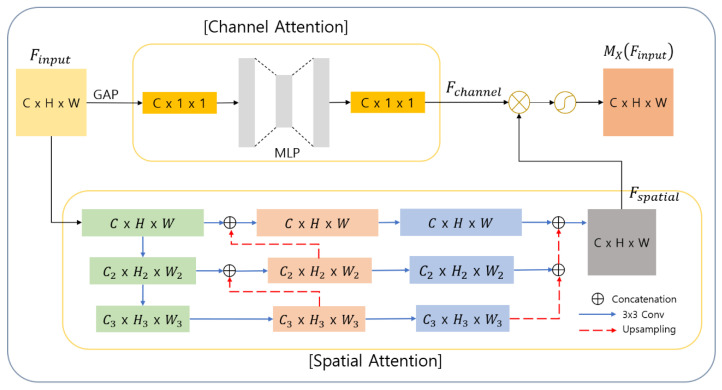
Structure of the X-attention module.

The structure of the X-attention module is shown in detail in Figure 1. The X-attention module focuses on the important features required for segmentation of lung areas by combining the features extracted through channel attention and spatial attention for the input feature map $F_{input}\;\in R^{C\;\times \;H\;\times \;W}$. The channel attention highlights the areas of the input feature maps corresponding to “what” in the learning process by readjusting the features between the channels of the input feature map. The core aspect of the channel attention in the X- and Y-attention modules is borrowed from the squeeze-and-excitation block [16]. However, for the rescaling step, the output of the spatial attention is used instead of the original input feature map. To calculate the channel attention, the input feature map ($F_{input}$) is compressed through global average pooling (GAP). The fully connected (FC) layer has been generally used to compress features. However, GAP was proposed to solve the problem of overfitting as well as the use of a large number of learning parameters, which are the common disadvantages of the FC layer. GAP is now increasingly used instead of the FC layer. Moreover, it can create compressed features that use global information more effectively because it uses the mean value of each channel of the input for compression. The compressed feature map ($\in R^{C\;\times \;1\;\times \;1}$) then passes through a multilayer perceptron (MLP), which consists of one hidden layer, to calculate the importance of each channel. The number of neurons of the hidden layer is Cr × 1 × 1, where r is a hyper-parameter to control a learnable parameter’s overhead. The output of the MLP is passed through batch normalization (BN) and the rectified linear unit function (ReLU), which is an activation function. The process of computing the channel attention map $F_{channel}\;\in \;R^{C\times 1\times 1}$ is shown in Equation (1).

(1) $$F_{channel}=ReLU\left(BN\left(MLP\left(GAP\left(F_{input}\right)\right)\right)\right)\;=ReLU\left(BN\left(W_{1}\left(W_{0}\left(MLP\left(GAP\left(F_{input}\right)\right)\right)\right)\right)\right),$$

where $W_{0}\in R^{C/r\;\times \;C}$ and $W_{1}\in R^{C\;\times \;C/r}$.

The spatial attention highlights the part of the feature maps corresponding to “where” in the learning process by combining the features extracted through multiple convolution layers. The input feature map ($F_{input}$) can generate feature maps of various scales by passing through 3 × 3 convolution layers consecutively. The intermediate feature maps are then transformed into the scale of the input feature map through up-sampling and are subsequently added together. For building the network for the spatial attention, we adopted the architecture of the Feature Pyramid Network (FPN) [21]. The FPN has been proven effective in extracting local information because it allows the network to learn with features of low to high levels through a structure for extracting and utilizing multiple feature maps of various scales. By using this structure, the spatial attention can effectively extract location information about important areas having important features that are required for segmentation. As the next step, the spatial attention map $F_{spatial}\;\in \;R^{C\times H\times W}$ is multiplied by the channel attention map $F_{channel}$ via element-wise multiplication. This multiplication generates the final attention map $M_{X}\in \;R^{C\times H\times W}$ of the X-attention module through the sigmoid function (σ), which is an activation function (see Equation (2)).

(2) $$M_{X}\left(F_{input}\right)=\;\sigma \left(\;F_{spatial}\;F_{channel}\right)$$

### 3.2. A Variant of the X-Attention Module (Y-Attention Module)

**Figure 2:**
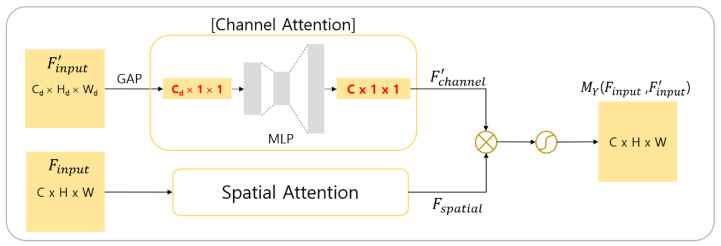
Structure of the Y-attention module.

In general, feature maps at shallow layers encode fine details, whereas feature maps at deeper layers carry more global semantic information. Therefore, we propose the Y-attention module to effectively utilize global features in input images. The proposed Y-attention module is a modified version of the X-attention module that accommodates the global context from a deeper layer. The key difference between the X-attention module and the Y-attention module is in the feature maps where the channel attention is extracted. The Y-attention module receives two feature maps, $F_{input}\in \;R^{C\times H\times W}$ and $F_{input}^{'}\;\in \;R^{C_{d}\times H_{d}\times W_{d}},$ as input. $F_{input}$ and $F_{input}^{'}$, which is an output of the deeper layer, are different feature maps of different scales. As $F_{input}^{'}$ contains global context information, it is used as an input to the channel attention. In addition, $F_{input}$ is used as an input of the spatial attention that can extract various local information because it is an output of the shallower layer. Note that as $F_{input}^{'}$ and $F_{input}$ are different feature maps, they may have a different number of channels. In this case, it is adjusted to the same number of channels as that of $F_{input}$ through MLP, which is used in the channel attention, to enable element-wise multiplication between attention maps. Finally, the attention map $M_{Y}\in \;R^{C\times H\times W}$ of the Y-attention module is generated through the sigmoid activation function (σ) (see Equation (3) and Figure 2).

(3) $$M_{Y}\left(F_{input},\;{F^{\prime }}_{input}\right)=\;\sigma \left(F_{spatial}⊗F^{\prime }{}_{channel}\;\right)$$

**Figure 3:**
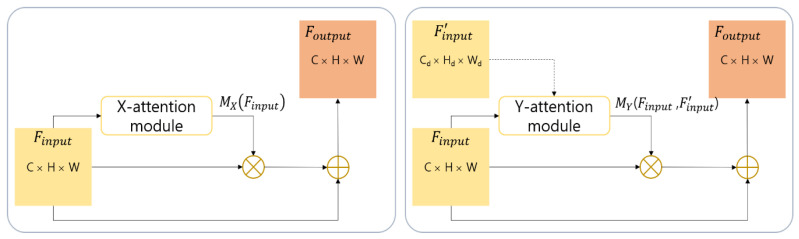
Residual learning structure of the X- and Y-attention modules.

Once the attention maps (e.g., $M_{X}\left(F_{input}\right)$ and $M_{Y}\left(F_{input},\;{F^{\prime }}_{input}\right)$) are successfully generated, the final refined feature maps are computed using a residual learning method (see Equations (4) and (5) and Figure 3). The residual learning scheme can realize improved performance without increasing the number of learnable parameters through a simple change of the deep learning network structure.

(4) $$F_{output}=\;M_{X}\left(F_{input}\right)⊗F_{input}\;+\;F_{input}$$

(5) $$F_{output}=\;M_{Y}\left(F_{input},F^{\prime }{}_{input}\right)⊗F_{input}\;+\;F_{input}$$

## 4. Results and Discussion

### 4.1. Datasets and Deep Neural Network Architecture

**Figure 4:**
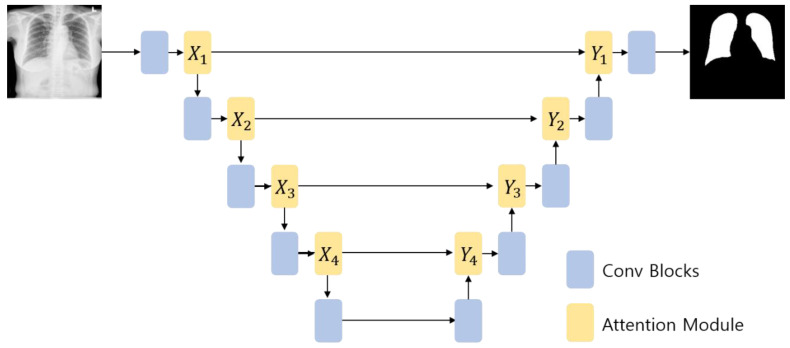
Example of the U-Net structure to which the X- and Y-attention modules were applied.

To assess the validity of the proposed attention module, experiments were conducted by applying it to U-Net, which is commonly used in the medical image segmentation field. U-Net was used in this experiment with ResNet101 as the backbone network. In addition, experiments were conducted while changing the position at which the attention module is placed in the U-Net structure (see Figure 4). According to the applied position, the attention modules are expressed as $X_{1}-Y_{4}$. For the implementation of U-Net and the attention module, the PyTorch framework was used.

We used X-ray images collected from three public datasets (the Montgomery dataset [22], the Japanese Society of Radiological Technology (JSRT) dataset [23], and the Shenzhen dataset [24]). The Montgomery dataset—published by the state health department of Montgomery, Alabama, in the U.S.—comprises a total of 138 images: 80 images of patients with tuberculosis and 58 images of people without disease. The JSRT dataset (released by the Japanese Society of Radiological Technology) comprises 154 images of patients with lung nodules and 93 images of people without disease. Lastly, the Shenzhen dataset contains X-ray images that have been collected from Shenzhen No. 3 Hospital of Guangdong Medical College, Shenzhen, China. The dataset contains 326 normal images and 336 abnormal images showing various manifestations of tuberculosis. 

In order to investigate the generalization capability of the proposed method, we used the datasets independently. For each dataset, data were randomly split into three subsets—training (70%), validation (10%), and testing (20%). For the final evaluation, we applied five-fold cross-validation on the dataset: for each fold, we measured the corresponding performance metric and the final result was the averaged result incorporating the standard deviation of the five evaluations. 

Prior to training and evaluation, images were resized to 512 × 512 pixels. The input images were not normalized but the brightness of the images was adjusted through histogram equalization. The lung segmentation result was also post-processed by removing areas other than the lung (the part excluding the largest two areas) to improve the image segmentation performance. The image segmentation performance was evaluated using the Dice score. In addition, the sensitivity and positive predictive value (PPV) were measured based on the image segmentation results. The corresponding equation is Equation (6), where TP, FP, and FN indicate the number of true positive, false positive, and false negative pixels, respectively.

(6) $$\mathrm{Dice}\;=\;\frac{2\cdot \mathrm{TP}}{\left(\mathrm{TP}+\mathrm{FP}\right)+\left(\mathrm{TP}+\mathrm{FN}\right)}\;\mathrm{Sensitivity}=\frac{\mathrm{TP}}{\mathrm{TP}\;+\mathrm{FN}}\;\mathrm{PPV}=\frac{\mathrm{TP}}{\mathrm{TP}+\mathrm{FP}}$$

### 4.2. Experimental Results and Discussion

The hyper-parameter values set for network training were as follows. The mini-batch size was set to four and the initial learning rate was set to 0.01. The learning rate was decreased by a factor of 10 when the validation set accuracy ceased to improve. Those values were empirically set. For the loss function, the mean squared error loss was used, and initial parameters that were trained in advance with ImageNet were used to accelerate the learning convergence. The whole network was optimized using the stochastic gradient descent function and the number of iterations for training was set to 10,000. However, we applied early stopping to avoid overfitting. We did not apply data augmentation to any of the deep learning models used in this study to observe the effects of the proposed attention modules. The reduction ratio used in the channel attention was set to 0.5 for both the X- and Y-attention modules. For training and testing, we used a single NVIDIA Titan-XP Graphics Processing Unit (GPU).

The number of learnable parameters in both the X- and Y-attention modules is very small compared to the baseline network (e.g., U-Net). For instance, the Y-attention module requires only five 3 × 3 filters to be learned. As a result, applying these attention modules in several places does not significantly increase the training time. Training the base U-Net required approximately four hours, whereas with the U-Net equipped with two X-attention modules and two Y-attention modules, approximately 1.5 to 2.5 h were required for training, depending on the amount of training data. The inference time was less than 1.4 s per chest X-ray image and the effect of adding attention modules was negligible. 

We conducted several lung segmentation experiments using chest X-rays while changing the locations of the proposed attention modules in the U-Net to assess the effectiveness of the attention modules. Table 1, Table 2 and Table 3 (the bold and underlined is best results) show the Dice score, sensitivity, and positive predictive value of the network structure according to the location of the attention modules, respectively. Note that U-Net + X(i)+ Y(j) indicates the combination of the X- and Y-attention modules with U-Net (i, j∈{1,2,3,4}). Compared to the cases where the X- and Y-attention modules were applied separately, their combined application showed slightly improved Dice scores. When only the X-attention module was applied, a small performance gain was observed when it was applied at the X(1) and X(2) positions compared to when it was applied at the X(3) and X(4) positions. This may be attributed to the fact that the attention modules at the X(1) and X(2) positions deal with feature maps in coarse scale and, therefore, help extract features for local image details. The same patterns appear with the Y-attention module; application of the Y-attention module at shallow layers performed slightly better than those at deeper layers.

**Figure 5:**
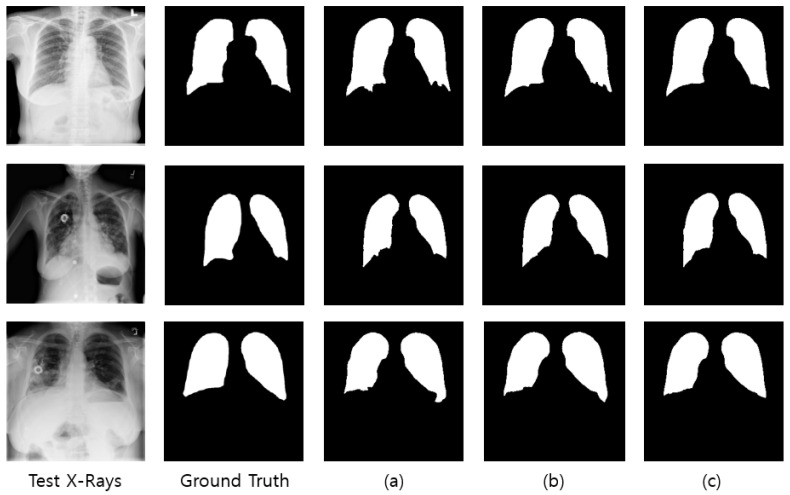
(**a**) Lung segmentation results of the U-Net + X(3)+ X(4)+ Y(3)+ Y(4) structure; (**b**) lung segmentation results of the U-Net + Y(1)+ Y(2)+ Y(3)+ Y(4) structure; (**c**) lung segmentation results of the U-Net + X(1)+ X(2)+ Y(1)+ Y(2) structure.

**Figure 6:**
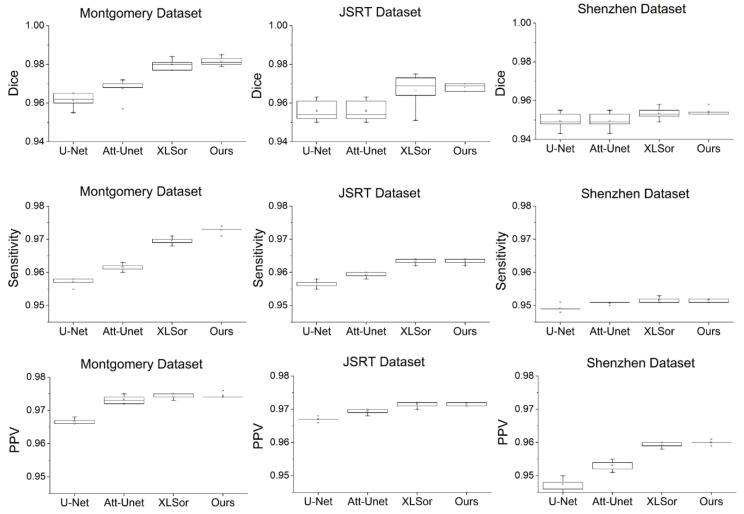
Comparative performance of lung segmentation on chest X-ray images.

**Figure 7:**
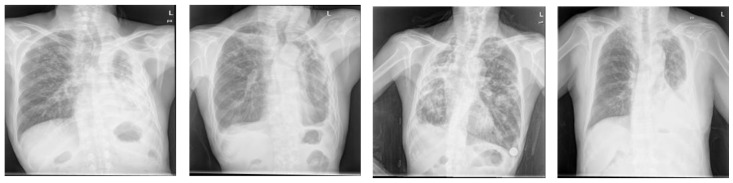
Hard examples with deformed lung shapes or ambiguous cardiac silhouettes contained in the Shenzhen dataset.

**Figure 8:**
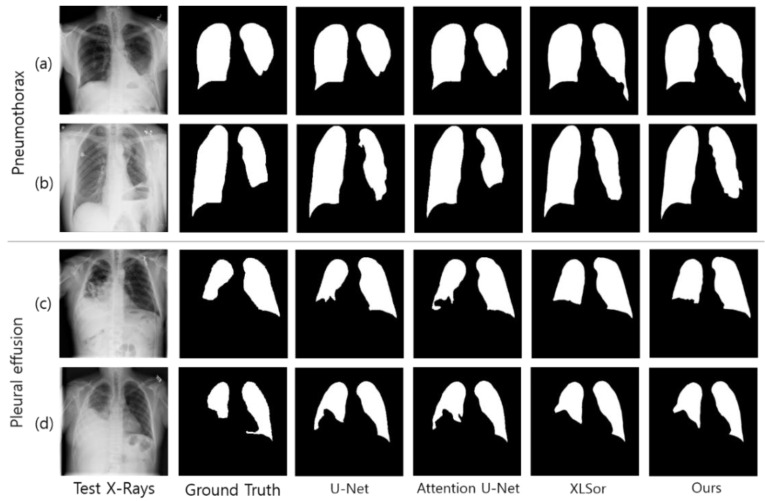
Chest X-ray and lung segmentation results of patients with pneumothorax (**a**,**b**) and pleural effusion (**c**,**d**).

When the network performance was compared by applying the attention module at various positions, the best segmentation performance was obtained when it was applied at the X(1)+X(2)+Y(1)+Y(2) position, irrespective of datasets used. With the Montgomery dataset and the JSRT dataset, the segmentation performance varies by up to 2.5% and 1.4%, respectively, depending on the locations of the X- and Y-attention modules, whereas the performance gain was marginal with the Shenzhen dataset. The performance improvement observed at the X(1)+X(2)+Y(1)+Y(2) position can be attributed to the fact that when the attention module is applied at the X(i)+ Y(i) (i∈{1,2,3,4}) position, the attention map extracted through X(i) is used as the input of the Y(i) attention module. The fine features can be highlighted by applying the attention modules consecutively rather than by applying X(i) and Y(j) (i, j∈{1,2,3,4}) separately. Furthermore, as the image segmentation must generate results with the same size as the input image, the X(i)+ Y(i) (i=1, 2) structure showed good performance. In general, initial layer features are typically more general whereas the latter layer features exhibit greater levels of specificity. Therefore, by locating attention modules in the initial layers, deep neural networks can take advantage of feature recalibration to improve the discriminative performance. This finding is consistent with the results of the empirical investigations conducted using SENet. Figure 5 illustrates examples of the chest X-rays and actual lung regions used for evaluation, as well as the lung segmentation images of three configurations that showed the best performance in the experimental results. On comparison of the Dice scores, the networks applying the proposed attention module achieved comparable or better performances than the other deep learning-based approaches (see Figure 6). When the Montgomery dataset or the JSRT dataset was used, the proposed method outperformed both standard U-Net and Attention U-Net. In the case of XLSor, however, both approaches showed similar segmentation performances but the proposed method worked relatively more stably. In the case of the Shenzhen dataset, although both XLSor and the proposed method achieved better performances than the standard U-Net and Attention U-Net, their performance gain was marginal. We conjectured that this result was attributed to high variability of lung segmentation masks due to the different lung shapes and borders in the Shenzhen dataset compared to the other two datasets [25]. In particular, the inclusion of X-ray images with deformed lungs or ambiguous cardiac silhouette as shown in Figure 7 will decrease the learning ability of deep learning models. The experimental results shown in Figure 8 also support this hypothesis.

**Figure 9:**
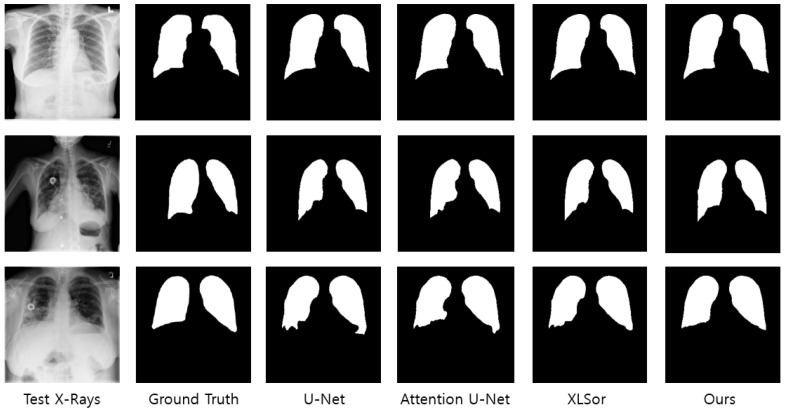
Performance comparison with existing deep learning models for lung segmentation.

Moreover, the network that applied the proposed attention module exhibited a deeper structure than the other networks because it uses U-Net based on ResNet and also performs residual learning for each attention map. A deep network may require a significantly longer learning time; however, important features can be effectively extracted in the learning process, thereby resulting in better performance when compared to existing networks. Figure 9 illustrates the segmentation results for the position (X(1)+X(2)+Y(1)+Y(2)) that exhibited the highest performance in the experiments and the segmentation results of the existing medical image segmentation networks. The networks that applied XLSor and the proposed attention module show relatively similar shapes to the lung area of the ground truth as compared to U-Net or Attention U-Net. Another observation is that the segmentation masks produced by our method are smooth with little noise. One possible reason is that the X- and Y-attention modules learned what and where to emphasize or suppress effectively, enabling them to provide accurate pixel-level attention information.

As observed in the aforementioned results, the proposed method showed good segmentation performance for chest X-ray images of normal lung shapes. However, deep learning-based methods, including the proposed method, exhibit poor segmentation performance for chest X-ray images with deformed lung shapes or lesions that obscure the border of heart and diaphragm, which was demonstrated in a previous study [4]. For instance, the chest X-rays of patients with pleural effusion do not have normal lung contours due to abnormal fluid accumulation. Moreover, in the chest X-ray image of a patient with pneumothorax, the presence of several holes in the lung alters the lung shape. Thus, a low segmentation performance is observed when chest X-ray images of these abnormal lung regions are used for evaluation (see Figure 8). To address such rare cases and improve the generalization capability of deep learning-based approaches, additional training datasets from such cases need to be used. 

## 5. Conclusions

This study proposed X- and Y-attention modules that can improve the performance of lung segmentation on chest X-ray images by highlighting fine features. The proposed attention module is composed of channel and spatial attention and enables the effective extraction of global and local features. The attention maps extracted through each attention modules are multiplied with each other and used as input for the next layer. To verify the validity of the attention module, experiments were conducted for various configurations of the attention module by combining it with U-Net. The experimental results suggest that the U-Net + X(1)+X(2)+Y(1)+Y(2) structure exhibits the highest segmentation performance among the various structures. Moreover, it showed comparable performance to XLSor, which is a state-of-the-art deep learning model for lung segmentation on chest X-ray images, on all of three public datasets, thus validating the method. However, chest X-rays with deformed lungs or ambiguous cardiac silhouette exhibited low lung segmentation performance, which remains a topic to be explored in future work. In addition, we plan to explore various tricks (including model tweaks, training refinements, data augmentation, and so on) to improve the generalization capability of the proposed deep learning model.

## Figures and Tables

**Table 1:** Comparison of Dice scores according to the location of the attention modules.

Configurations	Datasets		
	Montgomery	JSRT	Shenzhen
U-Net+X(1)	0.967 ± 0.002	0.959 ± 0.003	0.950 ± 0.001
U-Net+X(2)	0.967 ± 0.002	0.957 ± 0.002	0.949 ± 0.001
U-Net+X(3)	0.964 ± 0.002	0.956 ± 0.002	0.947 ± 0.002
U-Net+X(4)	0.962 ± 0.001	0.954 ± 0.001	0.947 ± 0.003
U-Net+Y(1)	0.964 ± 0.002	0.962 ± 0.002	0.950 ± 0.003
U-Net+Y(2)	0.960 ± 0.004	0.960 ± 0.002	0.947 ± 0.002
U-Net+Y(3)	0.959 ± 0.005	0.958 ± 0.002	0.947 ± 0.002
U-Net+Y(4)	0.957 ± 0.002	0.957 ± 0.008	0.947 ± 0.002
U-Net+X(1) +X(2)	0.970 ± 0.002	0.967 ± 0.001	0.950 ± 0.001
U-Net+X(3) +X(4)	0.965 ± 0.001	0.966 ± 0.001	0.947 ± 0.003
U-Net+X(1)+X(2)+X(3)+X(4)	0.970 ± 0.002	0.964 ± 0.003	0.947 ± 0.003
U-Net+Y(1) +Y(2)	0.969 ± 0.002	0.966 ± 0.003	0.951 ± 0.001
U-Net+Y(3) +Y(4)	0.966 ± 0.002	0.964 ± 0.003	0.947 ± 0.001
U-Net+Y(1) +Y(2)+Y(3) +Y(4)	0.968 ± 0.001	0.962 ± 0.001	0.950 ± 0.001
U-Net+X(1) +X(2)+Y(1) +Y(2)	**0.982 ± 0.002**	**0.968 ± 0.002**	**0.954 ± 0.002**
U-Net+X(3) +X(4)+Y(3) +Y(4)	0.972 ± 0.005	0.965 ± 0.001	0.949 ± 0.001

**Table 2:** Comparison of sensitivity according to the location of the attention modules.

Configurations	Datasets		
	Montgomery	JSRT	Shenzhen
U-Net+X(1)	0.971 ± 0.001	0.961 ± 0.001	0.949 ± 0.001
U-Net+X(2)	0.969 ± 0.001	0.960 ± 0.001	0.948 ± 0.001
U-Net+X(3)	0.966 ± 0.002	0.959 ± 0.001	0.948 ± 0.001
U-Net+X(4)	0.986 ± 0.001	0.958 ± 0.002	0.947 ± 0.001
U-Net+Y(1)	0.968 ± 0.001	0.959 ± 0.001	0.949 ± 0.001
U-Net+Y(2)	0.968 ± 0.001	0.959 ± 0.001	0.949 ± 0.001
U-Net+Y(3)	0.967 ± 0.001	0.959 ± 0.001	0.947 ± 0.001
U-Net+Y(4)	0.964 ± 0.002	0.958 ± 0.001	0.946 ± 0.001
U-Net+X(1) +X(2)	0.969 ± 0.002	0.960 ± 0.001	0.948 ± 0.001
U-Net+X(3) +X(4)	0.966 ± 0.001	0.959 ± 0.001	0.947 ± 0.001
U-Net+X(1)+X(2)+X(3)+X(4)	0.969 ± 0.002	0.961 ± 0.002	0.947 ± 0.001
U-Net+Y(1) +Y(2)	0.971 ± 0.001	0.962 ± 0.001	0.949 ± 0.001
U-Net+Y(3) +Y(4)	0.966 ± 0.002	0.958 ± 0.001	0.948 ± 0.001
U-Net+Y(1) +Y(2)+Y(3) +Y(4)	0.969 ± 0.002	0.959 ± 0.001	0.949 ± 0.001
U-Net+X(1) +X(2)+Y(1) +Y(2)	**0.973 ± 0.001**	**0.963 ± 0.001**	**0.952 ± 0.001**
U-Net+X(3) +X(4)+Y(3) +Y(4)	0.969 ± 0.001	0.959 ± 0.001	0.949 ± 0.001

**Table 3:** Comparison of positive predictive values according to the location of the attention modules.

Configurations	Datasets		
	Montgomery	JSRT	Shenzhen
U-Net+X(1)	0.971 ± 0.001	0.966 ± 0.001	0.956 ± 0.001
U-Net+X(2)	0.969 ± 0.001	0.965 ± 0.001	0.955 ± 0.001
U-Net+X(3)	0.965 ± 0.001	0.964 ± 0.001	0.955 ± 0.001
U-Net+X(4)	0.965 ± 0.002	0.965 ± 0.001	0.954 ± 0.001
U-Net+Y(1)	0.969 ± 0.001	0.965 ± 0.001	0.954 ± 0.002
U-Net+Y(2)	0.967 ± 0.001	0.966 ± 0.001	0.954 ± 0.001
U-Net+Y(3)	0.965 ± 0.001	0.965 ± 0.001	0.953 ± 0.001
U-Net+Y(4)	0.965 ± 0.001	0.965 ± 0.001	0.952 ± 0.001
U-Net+X(1) +X(2)	0.970 ± 0.001	0.968 ± 0.001	0.956 ± 0.001
U-Net+X(3) +X(4)	0.969 ± 0.001	0.966 ± 0.001	0.956 ± 0.001
U-Net+X(1)+X(2)+X(3)+X(4)	0.968 ± 0.001	0.966 ± 0.001	0.953 ± 0.001
U-Net+Y(1) +Y(2)	0.970 ± 0.001	0.966 ± 0.001	0.956 ± 0.001
U-Net+Y(3) +Y(4)	0.968 ± 0.001	0.965 ± 0.001	0.958 ± 0.003
U-Net+Y(1) +Y(2)+Y(3) +Y(4)	0.969 ± 0.001	0.966 ± 0.001	0.956 ± 0.001
U-Net+X(1) +X(2)+Y(1) +Y(2)	**0.974 ± 0.001**	**0.971 ± 0.001**	**0.960 ± 0.001**
U-Net+X(3) +X(4)+Y(3) +Y(4)	0.967 ± 0.001	0.967 ± 0.001	0.956 ± 0.001

## Data Availability

Source codes and learned models are available from the authors upon reasonable request.
